# Draft Genome Sequences of Two *Clostridium* Isolates from the Poultry Gastrointestinal Tract

**DOI:** 10.1128/MRA.00137-20

**Published:** 2020-05-28

**Authors:** Jitendra Keshri, Rocio Ramirez, Raja Chalghoumi, Johnna K. Garrish, Bruce S. Seal, Brian B. Oakley

**Affiliations:** a College of Veterinary Medicine, Western University of Health Sciences, Pomona, California, USA; b Graduate College of Biomedical Sciences, Western University of Health Sciences, Pomona, California, USA; c Animal Science Department, College of Agriculture of Mateur, University of Carthage, Mateur, Bizerte, Tunisia; d Poultry Microbiological Safety and Processing Research Unit, USDA, Agricultural Research Service, U.S. National Poultry Center, Athens, Georgia, USA; e Biology Program, Oregon State University Cascades, Bend, Oregon, USA; University of Arizona

## Abstract

Here, we announce the draft genome sequences of two *Clostridium* strains, C8-1-8 and C2-6-12, isolated from the cecal contents of commercial broiler chickens (in Athens, GA). These strains may represent potentially novel species within the genus *Clostridium*, and these draft genomes allow further investigation into potential probiotics for poultry.

## ANNOUNCEMENT

The taxonomy and systematics of the genus *Clostridium* are complex and unresolved, but a total of 231 recognized species belonging to the genus *Clostridium* were reported as of 2019 (http://www.bacterio.net/clostridium.html). We isolated two *Clostridium* strains, designated strains C8-1-8 and C2-6-12, from the ceca of commercial broiler chickens collected in Athens, Georgia. These strains may represent potentially novel species, as suggested by a comparison based on whole-genome sequencing. The strains were isolated from cecal contents treated with 3% chloroform to eliminate vegetative bacterial cells (1, 2) and cultured in an anaerobic environment on gut microbiota medium (3). Single colonies were subsequently propagated overnight in Luria-Bertani broth for DNA extraction from cultures using the MO BIO UltraClean microbial DNA isolation kit. Initial characterizations of the isolates were completed by Gram staining followed by 16S rRNA gene sequence analyses. The genomes of both strains were sequenced using the Illumina NovaSeq platform through the Institute for Integrative Genome Biology Genomics Core, University of California, Riverside.

Libraries with a fragment length of 150 bp were constructed using the plexWell 96 library preparation kit for Illumina sequencing platforms (seqWell, Inc., Beverly, MA, USA), and totals of 9,074 and 9,415 Mb of paired-end (150-bp) reads were generated for strains C8-1-8 and C2-6-12, respectively. Default parameters for all software were used during analyses unless otherwise specified. Quality filtering of the reads was performed using Trimmomatic v0.39 (4). High-quality reads were used for *de novo* genome assembly with SPAdes v3.12.0 using the careful assembly method (5). Scaffolds were filtered for a minimum of 200-bp read length and 30× coverage. The quality of the subsequent assemblies was assessed using QUAST (6). Genome completeness was found to be 99.1% for both strains, using the CheckM lineage (7) and Microbial Genomes Atlas (MiGA) (http://microbial-genomes.org) (8) workflows. The species identification tool specI, based on 40 universal single-copy marker genes (9), confirmed that both genomes could not be assigned to any species cluster. Similarly, neither genome could be assigned to a species when compared against reference genomes from the NCBI RefSeq database using MiGA (8). The results of assembly quality analysis by the MiGA workflow based on 106 essential genes were rated as intermediate and high for C8-1-8 and C2-6-12, respectively. The assembled genomes were annotated using the IMG/MER pipeline (10), and average coverage was calculated using the BBMap tool. Assembly and annotation metrics are presented in Table 1.

**TABLE 1:** Assembly and annotation metrics

Statistic	Data for strain:	
	C8-1-8	C2-6-12
No. of quality-filtered paired-end reads	11,102,701	10,624,933
No. of scaffolds	116	121
*N*_50_ (bp)	177,332	126,571
Largest scaffold length (bp)	442,315	352,209
Assembly length (bp)	6,053,167	6,076,981
Avg genome coverage (×)	448	471
G+C content (%)	31.6	29.02
No. of rRNA genes	14	10
No. of tRNA genes	47	57
No. of functional protein-coding genes	3,956	4,254
No. of hypothetical protein-coding genes	1,413	1,373
No. of genes associated with KEGG pathways	1,209	1,354
No. of genes associated with KEGG orthology	3,091	2,350
GenBank accession no.	JAABNO000000000	JAABNP000000000
SRA accession no.	SRR10903102	SRR10903103

Based on the compare genome analysis function in IMG, which uses the genome-wide average nucleotide identity (ANI), the maximum pairwise ANI of strain C8-1-8 with available *Clostridium* genomes (*n* = 756) was 92.6% with *Clostridium* sp. strain CT4 (GenBank accession number CP025746; aligned fraction [AF], 79.7%). The maximum ANI of strain C2-6-12 with known *Clostridium* genomes was 84.8% with Clostridium puniceum DSM 2619 (GenBank accession number LZZM00000000.1; IMG genome identification number 2703719317; AF, 53.8%). Comparing our genomes to all available genomes of *Clostridium* type strains (*n* = 80), the maximum ANI for strain C8-1-8 was 73.3% with Clostridium fallax DSM 2631 (AF, 17.8%) and the maximum ANI for strain C2-6-12 was 84.8% (AF, 53.8%) with *C. puniceum* BL70/20. The ANI between our two isolates was only 72.57%, suggesting that strains C8-1-8 and C2-6-12 are distantly related to each other.

### Data availability.

The draft genome and raw read sequences for both strains have been deposited in DDBJ/EMBL/GenBank under the accession numbers listed in Table 1.
